# Severity of symptoms, physical functioning and satisfaction in patients with lumbar spinal stenosis: a validation study of the Iranian version of the Swiss Spinal Stenosis Score (SSS)

**Published:** 2012-11

**Authors:** Parisa Azimi, Sohrab Shahzadi, Hossein Safdari Ghandehari, Sohrab Sadeghi, Shirzad Azhari, Hossein Nayeb Aghaei, Hassan Reza Mohammadi, Ali Montazeri

**Affiliations:** ^*a*^Department of Neurosurgery, Shahid Beheshti University of Medical Sciences, Tehran, Iran.; ^*b*^Mental Health Research Group, Health Metrics Research Centre, Iranian Institute for Health Sciences Research, ACECR, Tehran, Iran.

## Abstract

**Background:**

A common cause of low back pain is lumbar spinal stenosis (LSS). The Swiss Spinal Stenosis Score (SSS) is a well-known questionnaire measuring the severity of symptoms, physical functioning and patient’s satisfaction in these patients. This study aimed to translate and validate the SSS in Iran.

**Methods:**

This was a prospective clinical validation study. Forward-backward procedure was applied to translate the original questionnaires into Persian. A sample of patients with lumbar spinal stenosis completed the questionnaire twice: pre- and post-operative (6-month follow-up) assessments. To test reliability the internal consistency was assessed; Validity was evaluated using known groups comparison. In addition Oswestry Disability Index was used to perform convergent validity.

**Results:**

In all 121 patients were entered into the study. The mean age of patients was 62.3 (SD equal to 10.2) years. Cronbach’s alpha coefficient for the SSS was found to be 0.88. Validity assessment was performed using common group’s analysis and showed satisfactory results. The instrument discriminated efficiently between sub-groups of patients who differed in age, severity of lumbar spinal stenosis, and Self-Paced Walking Test (SPWT). The change in Oswestry Disability Index strongly correlated with the change in patients’ scores on the SSS, lending support to its good convergent validity (r equal to 0.82; P Less than 0.001).

**Conclusions:**

The Iranian version of the Swiss Spinal Stenosis Score performed well and the findings suggest that it is a valid measure of the severity of symptoms, physical function and satisfaction among lumbar spinal stenosis patients.

**Keywords:**

Lumbar, Spinal stenosis, Swiss Spinal Stenosis Score


					Volume 4, Suppl. 1 Nov 2012
Publisher: Kermanshah University of Medical Sciences
URL: http://www.jivresearch.org
Copyright:
Congress of Iranian Neurosurgeons, 14-16 November 2012, Kermanshah, Iran
Paper No. 76
Severity of symptoms, physical functioning and satisfaction
in patients with lumbar spinal stenosis: a validation study of
the Iranian version of the Swiss Spinal Stenosis Score (SSS)
Parisa Azimi a,*
, Sohrab Shahzadi a
, Hossein Safdari Ghandehari a
, Sohrab Sadeghi a
, Shirzad Azhari a
,
Hossein Nayeb Aghaei a
, Hassan Reza Mohammadi a
, Ali Montazeri b
a
Department of Neurosurgery, Shahid Beheshti University of Medical Sciences, Tehran, Iran.
b
Mental Health Research Group, Health Metrics Research Centre, Iranian Institute for Health Sciences Research, ACECR,
Tehran, Iran.
Abstract:
Background: A common cause of low back pain is lumbar spinal stenosis (LSS). The Swiss Spinal Stenosis Score (SSS)
is a well-known questionnaire measuring the severity of symptoms, physical functioning and patient's satisfaction in
these patients. This study aimed to translate and validate the SSS in Iran.
Methods: This was a prospective clinical validation study. Forward-backward procedure was applied to translate
the original questionnaires into Persian. A sample of patients with lumbar spinal stenosis completed the questionnaire
twice: pre- and post-operative (6-month follow-up) assessments. To test reliability the internal consistency was
assessed; Validity was evaluated using known groups comparison. In addition Oswestry Disability Index was used to
perform convergent validity.
Results: In all 121 patients were entered into the study. The mean age of patients was 62.3 (SD equal to 10.2)
years. Cronbach's alpha coefficient for the SSS was found to be 0.88. Validity assessment was performed using
common group's analysis and showed satisfactory results. The instrument discriminated efficiently between sub-groups
of patients who differed in age, severity of lumbar spinal stenosis, and Self-Paced Walking Test (SPWT). The change
in Oswestry Disability Index strongly correlated with the change in patients' scores on the SSS, lending support to its
good convergent validity (r equal to 0.82; P Less than 0.001).
Conclusions: The Iranian version of the Swiss Spinal Stenosis Score performed well and the findings suggest that it is
a valid measure of the severity of symptoms, physical function and satisfaction among lumbar spinal stenosis patients.
Keywords:
Lumbar, Spinal stenosis, Swiss Spinal Stenosis Score
* Corresponding Author at:
Parisa Azimi: Department of Neurosurgery, Imam-Hossain Hospital, Shahid-Beheshti University of Medical Sciences, Imam-Hossain sq., Tehran, Iran.
Email: Parisa.azimi@gmail.com, (Azimi P.).

				

